# Mixed ganglioneuroma and cortical adenoma in adrenal gland: A case report

**DOI:** 10.1097/MD.0000000000031403

**Published:** 2022-11-25

**Authors:** Yoo Na Kang

**Affiliations:** a Department of Forensic Medicine, Kyungpook National University School of Medicine, Daegu, Republic of Korea.

**Keywords:** adrenal gland, cortical adenoma, corticomedullary tumor, ganglioneuroma, mixed

## Abstract

Adrenal mixed corticomedullary tumors (MCMTs) are single tumor masses composed of an intimately admixed population of both adrenal cortical cells and medullary components. Most medullary tumor components are pheochromocytomas; however, MCMTs composed of ganglioneuroma and adrenal cortical adenoma are extremely rare. The current case is a rare case of adrenal MCMT composed of ganglioneuroma and adrenal cortical adenoma with primary aldosteronism.

**Patient concerns:**

A 49-year-old male was admitted because of hypokalemia and an adrenal mass. He was diagnosed with hypertension in his 20s and was taking blood pressure medications.

**Diagnosis:**

Plasma aldosterone concentration 376.5 pg/dL (normal 37.8~233.0 pg/mL) and potassium 2.8 mmol/L (normal 3.4~4.9 mmol/L) were detected. The aldosterone-to-renin ratio [the ratio of plasma aldosterone concentration (ng/dL) to PRA (ng/mL/hour)] was 38. The saline loading test showed that serum aldosterone (49.4 ng/dL) was not suppressed, compared with the basal level (28.4 ng/dL). The adrenal venous sampling test showed that the aldosterone level markedly increased to 1521.2 pg/mL. Abdominal computed tomography revealed an enlarged relatively well-circumscribed multinodular mass (35 × 13 × 30 mm) in the right adrenal gland.

**Interventions:**

Laparoscopic right adrenalectomy was performed under the clinical diagnosis of a functioning adrenal cortical adenoma.

**Outcomes:**

After laparoscopic right adrenalectomy, the serum aldosterone and renin levels returned to normal. The patient maintained a normal aldosterone level without recurrence for 16 months.

**Lessons:**

Adrenal MCMTs of the ganglioneuroma and cortical adenomas in the ipsilateral adrenal gland are extremely rare. Adrenal MCMTs exhibit benign clinical behavior, with no metastasis or death due to the tumor. With the development of diagnostic imaging technology, it is possible to identify mixed tumors. However, surgical resection of adrenal gland is a common treatment and a final diagnosis should be made based on the pathological results after surgery. Because this is to rule out the occurrence of rare malignant tumors and confirm the pattern of mixed tumors.

## 1. Introduction

Ganglioneuroma (GN) is an asymptomatic benign tumor that occurs along the paravertebral sympathetic ganglia and rarely occurs in the adrenal medulla. Owing to its asymptomatic and hormonally silent nature, most cases are incidentally detected during imaging for unrelated reasons. Adrenal cortical adenoma, which is considered to be the most common component, is usually nonfunctional. However, when the tumor is functional, increases in testosterone, cortisol, and aldosterone may be induced.

Adrenal mixed tumors comprise 2 histologically distinct adrenal tumors with a low incidence rate. Depending on whether it is an adrenal cortical or medullary component, adrenal mixed tumors are divided into composite tumors and mixed corticomedullary tumors (MCMTs).

MCMT is an even rarer form and is composed of both adrenal cortical cells and medullary tumors. Here we report a rare case of a mixed adrenal tumor composed of functioning adrenal cortical adenoma and GN.

## 2. Case report

**Figure 1.**
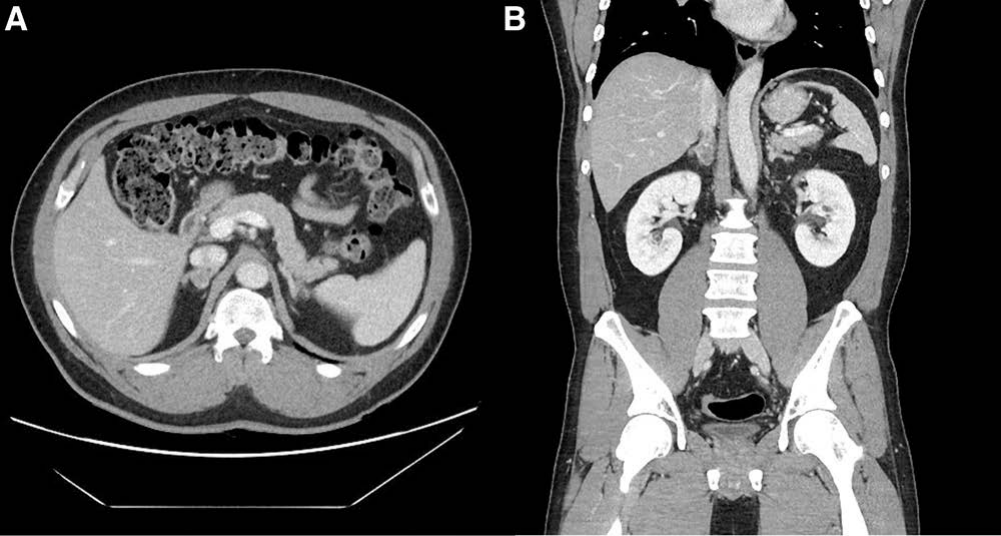
Abdominal CT reveals a 3.5 cm-sized, well-circumscribed, lobulated mass in the right adrenal gland, (A) white arrow on axial image and (B) black arrow on coronal image). On enhanced CT scan, it shows heterogeneous enhancement with punctate calcifications. CT = computed tomography.

A 49-year-old male presented to the hospital with gastrointestinal symptoms. On physical examination, the patient’s condition was normal. During the hospital examination, hypokalemia and adrenal mass were incidentally discovered. In his 20s, he was diagnosed with hypertension and taking blood pressure medication. An aldosterone test was performed to determine the cause of hypokalemia. Plasma aldosterone concentration 376.5 pg/dL (normal 37.8~233.0 pg/mL), plasma renin activity (PRA) 0.9 ng/mL/hour (normal 0.6 to 4.3 ng/mL/hour), sodium 142 mmol/L (normal 136~145 mmol/L), potassium 2.8 mmol/L (normal 3.4~4.9 mmol/L) were detected. The aldosterone-to-renin ratio, the ratio of plasma aldosterone concentration (ng/dL) to PRA (ng/mL/hour), was 38. The saline loading test showed that serum aldosterone (49.4 ng/dL) was not suppressed, compared with the basal level (28.4 ng/dL). The adrenal venous sampling test showed that the aldosterone level markedly increased to 1521.2 pg/mL. Abdominal computed tomography (CT) reveals a 3.5 cm-sized, well-circumscribed, lobulated mass with heterogeneous enhancement and punctate calcification in the right adrenal gland (Fig. 1). Among variable lesions that form adrenal tumor, such as adrenal adenoma, myelolipoma, lipoma, pheochromocytoma, adrenal cancer, and metastatic cancer, an adrenal adenoma with aldosterone secretion was suspected.

**Figure 2.**
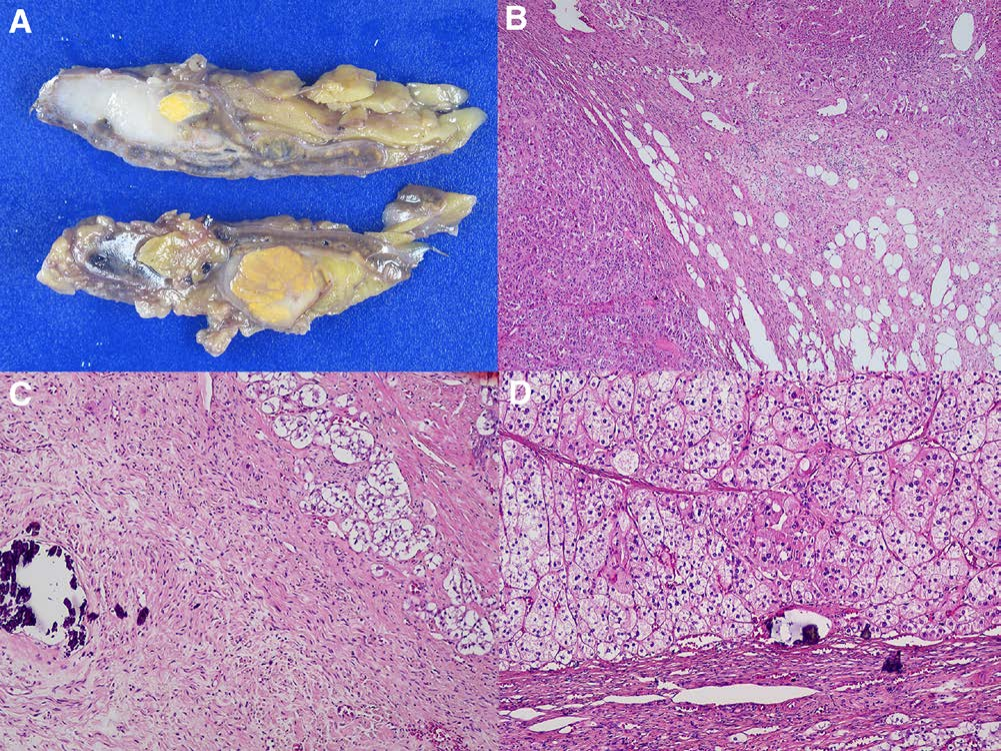
Cut section of right adrenal gland shows an enlarged relatively well-circumscribed multinodular mass, measuring 35 × 13 × 30 mm (A). An elongated grayish-white mass is clearly demarcated from a round yellow mass (A). The borderline between 2 different tumor masses are also distinct, microscopically (H&E, ×40) (B). In the low power view, the grayish-white portion (right) shows wavy spindle cells are intermixed with adipose tissue (B). GN portion of grayish-white mass is composed of numerous wavy spindle Schwann cells, few scattered eosinophilic mature ganglion cells, and dystrophic calcification (H&E, ×200) (C). Adrenal cortical adenoma portion of round yellow mass shows diffusely homogeneous tumor nests composed of abundant foamy cytoplasm and a few atypical nuclei with dystrophic calcification (H&E, ×100) (D). GN = Ganglioneuroma.

A laparoscopic right adrenalectomy was performed with potassium supplementation and anti-aldosterone drug administration. A cut section of the right adrenal gland showed an enlarged, relatively well-circumscribed, multinodular mass measuring 35 × 13 × 30 mm. An elongated grayish-white mass was clearly demarcated from the round yellow mass (Fig. 2A). The borderline between the 2 tumor masses was microscopically distinct (Fig. 2B). The GN portion of the grayish-white mass was composed of numerous wavy spindle Schwann cells, a few scattered eosinophilic mature ganglion cells, and dystrophic calcification (Fig. 2C). The adrenal cortical adenoma portion of the round yellow mass showed diffusely homogeneous tumor nests composed of abundant foamy cytoplasm and a few atypical nuclei with dystrophic calcifications (Fig. 2D). No evidence of abnormal mitosis, hemorrhage or necrosis was observed in any portion of the tumor mass.

**Figure 3.**
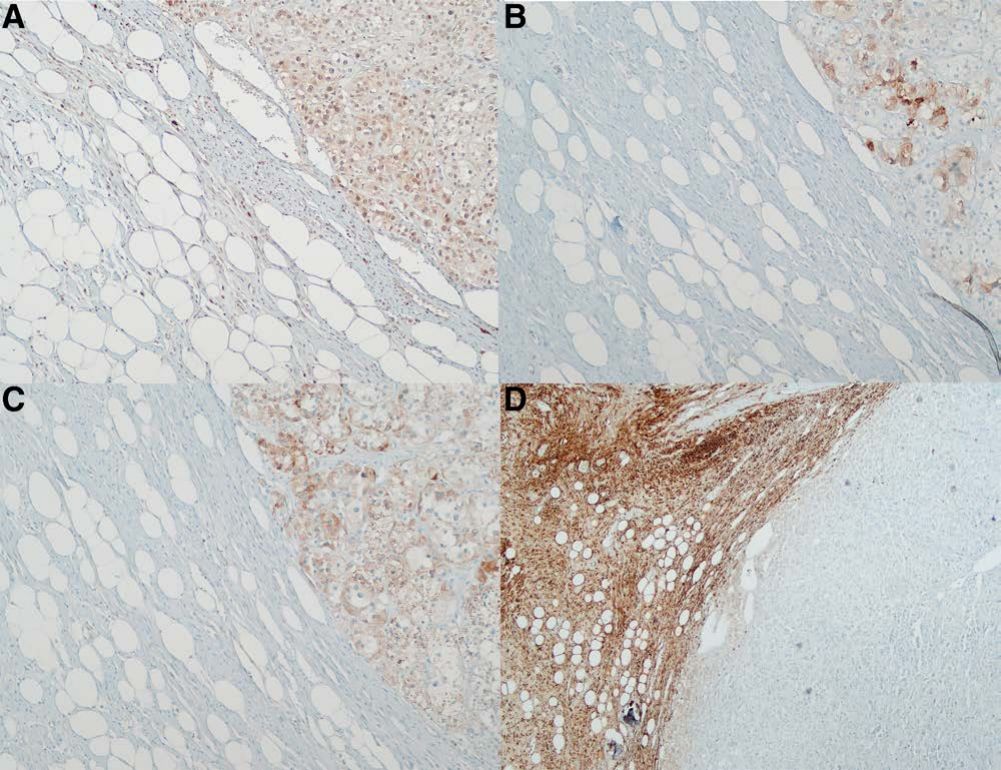
Immunohistochemical stains of right adrenal tumor shows positive reaction in the cortical adenoma portion (right side of each figures) for calretinin (A, ×100), inhibin (B, ×100) and Melan-A(C, ×100), while negative in the GN portion (left side of each figures). The GN portion shows diffuse strong positivity for S100 (D, ×40). GN = Ganglioneuroma.

In the cortical adenoma portion, calretinin, inhibin, and Melan-A showed positive reactions. The GN portion showed a strong diffuse positivity for S100 (Fig. 3). Therefore, the patient was diagnosed with a MCMT comprising adrenal cortical adenoma and GN in the ipsilateral adrenal gland. After laparoscopic right adrenalectomy, the serum aldosterone and renin levels returned to normal. The patient maintained normal aldosterone levels without recurrence until 16 months after the surgery. The patient is managing blood pressure while using calcium channel blockers and angiotensin II receptor blockers.

## 3. Discussion

GNs are rare, benign, differentiated tumors that are composed of mature Schwann cells, nerve fibers, and a variable number of ganglion cells.^[1]^ They occur in the posterior mediastinum and retroperitoneum, with only a minor proportion occurring in adrenal glands.^[2]^ Adrenal cortical adenomas are the most common tumors in the adrenal cortex. They are usually nonfunctional, but can sometimes be detected by hormonal symptoms, such as Cushing’s syndrome, hyperaldosteronism, or virilization.^[1]^

The adrenal cortex and medulla have different embryological origins and produce different hormones.^[2]^ Adrenal neoplasms may be composed of more than 1 cell type and may exhibit a mixed histological appearance. These are called composite tumors and MCMTs, respectively.

A composite tumor is composed of more than 1 medullary-type tumor, including endocrine and neural elements. Composite pheochromocytoma comprises pheochromocytoma and other medullary tumors, including malignant peripheral nerve sheath tumors, neuroendocrine-neural tumors, GN, ganglio-neuroblastoma, neuroblastoma, and spindle cell sarcoma.^[3–5]^ For example, composite pheochromocytoma/GN arises in the background of diffuse and nodular medullary hyperplasia of the adrenal gland.^[3]^ Tumor cells were histologically classified as chromaffin- or chromaffin-like, neuron-like, or intermediate. These cell types may represent a spectrum of differentiation in a neoplastic clone, with intermediate cells representing a transitional stage between the chromaffin cells and neurons.^[3]^

A MCMT represents an even rarer form of compound adrenal gland tumor. MCMT is characterized by a single mass composed of both adrenal cortical and medullary cells. Among the MCMTs, most medullary tumors are pheochromocytomas.^[6,7]^ But, the occurrence of synchronous GN with cortical adenoma in the ipsilateral adrenal gland is exceedingly rare. To the best of our knowledge, only 6 cases including the current case, have been reported to date (Table 1).

**Table 1:** Reported cases of mixed tumors of GN and adrenal cortical adenoma.

**Year**	**Study**	**Age/Sex**	**Tumor size**	**Diagnosis**	**Clinical Menifestations**
1978	Takahashi^[8]^	38/F	16.0 cm	Mixed GN and adreno-cortical adenoma	High level of serum testosterone
1988	Aiba^[9]^	53/M	5.3 cm	Compound adrenal medullary tumor (pheo and GN) and cortical adenoma	Cortisol-producing
2005	Bernini^[10]^	69/F	2.5 cm	Pheo, GN, adrenal cortical adenoma, hepatic and vertebral hemangiomas	nonfunctioning
2006	Rodriguez-Justo^[2]^	44/M	4.5 cm	Adrenal cortical adenoma and GN	Normal levels of aldosterone and renin
2011	Lau^[11]^	64/F	3.6 cm	Mixed cortical adenoma and composite pheo-GN	Hypertension
2022	Current case	49/M	3.5 cm	Mixed GN and adreno-cortical adenoma	Aldosteronism, Hypertension

GN = Ganglioneuroma, Pheo = Pheochromocytoma.

Takahashi et al presented a gonadotrophin-responsive virilizing adrenal tumor identified as a mixed GN and adrenal cortical adenoma in 1978. The adrenal mass was a circumscribed irregularly lobulated mass that was histologically GN containing numerous scattered groups of large polyhedral cells. After removal of the right adrenal tumor, the high serum testosterone level decreased to normal within 24 hours.^[8]^ Aiba et al presented a compound adrenal medullary tumor (pheochromocytoma and GN) and cortical adenoma in the ipsilateral adrenal gland, suggesting that the adenoma was cortisol-producing.^[9]^ Simultaneous occurrence of adrenal cortical adenoma and GN with normal levels of aldosterone and renin, was reported by Rodriguez-Justo et al^[2]^ Lau presented with an unusual MCMT of the adrenal gland (mixed cortical adenoma and composite pheochromocytoma-GN) with a history of hypertension for 15 years, controlled with medication.^[11]^

In these 6 adrenal MCMTs with GN, cortical and medullary tumor components in 2 cases were found separately in the ipsilateral adrenal gland.^[2,9]^ Three MCMT cases had composite pheochromocytoma-GN as medullary tumor with cortical adenoma.^[9–11]^ These included 3 males and 3 females. Age ranged from 38 to 69 years old (mean 52.8 years old). The tumor diameter was 2.5 to 16.0 cm (mean 5.9 cm). There was 1 case each with increased testosterone, cortisol and aldosterone levels, and 2 patients had a history of hypertension (Table 1).

According to a report of 80 patients with GN alone, there were 36 males and 44 females. The mean age at onset was 37.7 years, and the mean tumor was 4.3 cm.^[12]^ Compared to GN alone, MCMTs occur at an older age and have a larger tumor size. In addition, malignant MCMTs occurred at the age of 63 and 78 years and occurred at an older age than MCMT.^[13,14]^ According to the MCMT literature review, total MCMTs regardless of GN or Pheochromocytoma as a medullary tumor were found almost exclusively in females. In the vast majority of patients, symptoms are related to hormone hypersecretion in the tumor. Hypertension and diabetes were present in 80 and 40% of cases, respectively. Cushing’s syndrome was reported in 53.33% of cases.^[14]^

Most adrenal lesions are benign and are detected incidentally due to the absence of symptoms. The prevalence of incidental adrenal lesions has been reported to be 2.3% at autopsy and 0.5% to 2% on abdominal CT.^[15]^ Nevertheless, the probability of an adrenal mass being a metastasis is high in patients with an extra-adrenal primary cancer. Although recent advances in CT and magnetic resonance imaging have aided the identification of benign and malignant adrenal lesions, there may be a significant overlap between the imaging appearances of benign lesions, such as lipid-poor adenomas, and malignant lesions. Moreover, malignant MCMTs have recently been reported.^[13,14]^ Therefore, diagnostic confirmation after surgical resection of adrenal mass is necessary in combination with biochemical and clinical data as well as radiologic evaluation. In the case of adrenal GN, laparoscopic surgery has become the current method for resecting tumors owing to the advantages of small trauma and rapid recovery. No recurrence or metastasis after GN resection was observed during the follow-up period of maximum 35 years.^[13]^

For the differential diagnosis of adrenal neoplasms with mixed histological appearance, immunohistochemical stains supporting the morphologic impression of a tumor with mixed cortical medullary and neural features may be needed. The cortical cells exhibited positive reactions for alpha-inhibin and Melan A, and the pheochromocytes were positive for chromogranin A and synaptophysin, with S100-positive sustentacular cells. In GN, the spindle Schwann cell component showed positive expression of S100 protein, whereas ganglion cells were positive for synaptophysin and neurofilament proteins. All 6 MCMT with GN patients including the current case underwent adrenalectomy and were confirmed by histological examination with immunohistochemical stains.

In conclusion, adrenal MCMTs composed of GN and cortical adenoma are extremely rare, although composite tumors composed of more than 1 type of medullary tumor in the adrenal gland often occur. Adrenal MCMTs exhibit benign clinical behavior, with no metastasis or death due to the tumor. With the development of diagnostic imaging technology, it is possible to identify mixed tumors. However, surgical resection is a common treatment and a final diagnosis should be made based on the pathological results after surgery. Because this is to rule out the occurrence of rare malignant tumors and to confirm the pattern of mixed tumors. At the same time, it is meaningful to accumulate clinical and pathological data on very rare adrenal MCMT (GN and cortical adenoma) to characterize and treat MCMT.

## Author contributions

**Writing – original draft:** Yoo Na Kang.

## References

[1] Lee HS Choi YJ Kim C. Adrenal collision tumor: coexistence of pigmented adrenal cortical oncocytoma and ganglioneuroma. Case Rep Surg. 2016;2016:5790645. 28053800 10.1155/2016/5790645 PMC5178330

[2] Rodriguez-Justo M Chan G. Simultaneous occurrence of adrenal cortical adenoma and ganglioneuroma. Histopathology. 2006;49:206–8. 16879403 10.1111/j.1365-2559.2006.02384.x

[3] Brady S Lechan RM Schwaitzberg SD. Composite pheochromocytoma/ganglioneuroma of the adrenal gland associated with multiple endocrine neoplasia 2A: case report with immunohistochemical analysis. Am J Surg Pathol. 1997;21:102–8. 8990146 10.1097/00000478-199701000-00011

[4] Okumi M Ueda T Ichimaru N. A case of composite pheochromocytoma-ganglioneuroblastoma in the adrenal gland with primary hyperparathyroidism. Hinyokika Kiyo. 2003;49:269–72. 12822455

[5] Tatekawa Y Muraji T Nishijima E. Composite pheochromocytoma associated with adrenal neuroblastoma in an infant: a case report. J Pediatr Surg. 2006;41:443–5. 16481267 10.1016/j.jpedsurg.2005.11.024

[6] Chu AY LiVolsi VA Fraker DL. Corticomedullary mixed tumor of the adrenal gland with concurrent adrenal myelolipoma. Arch Pathol Lab Med. 2003;127:e329–32. 12873195 10.5858/2003-127-e329-CMTOTA

[7] Lee P Bradbury RA Sy J. Phaeochromocytoma and mixed corticomedullary tumour - a rare cause of Cushing’s syndrome and labile hypertension in a primigravid woman postpartum. Clin Endocrinol (Oxf). 2008;68:492–4. 17868399 10.1111/j.1365-2265.2007.03038.x

[8] Takahashi H Yoshizaki K Kato H. A gonadotrophin-responsive virilizing adrenal tumour identified as a mixed ganglioneuroma and adreno-cortical adenoma. Acta Endocrinol (Copenh). 1978;89:701–9. 213919 10.1530/acta.0.0890701

[9] Aiba M Hirayama A Ito Y. A compound adrenal medullary tumor (pheochromocytoma and ganglioneuroma) and a cortical adenoma in the ipsilateral adrenal gland. A case report with enzyme histochemical and immunohistochemical studies. Am J Surg Pathol. 1988;12:559–66. 3389453 10.1097/00000478-198807000-00008

[10] Bernini G Moretti A Mannelli M. Unique association of non-functioning pheochromocytoma, ganglioneuroma, adrenal cortical adenoma, hepatic and vertebral hemangiomas in a patient with a new intronic variant in the VHL gene. J Endocrinol Invest. 2005;28:1032–37. 16483185 10.1007/BF03345345

[11] Lau SK Chu PG Weiss LM. Mixed cortical adenoma and composite pheochromocytoma-ganglioneuroma: an unusual corticomedullary tumor of the adrenal gland. Ann Diagn Pathol. 2011;15:185–9. 20952294 10.1016/j.anndiagpath.2010.02.005

[12] Fan H Li HZ Ji ZG. Diagnosis and treatment of adrenal ganglioneuroma: a report of 80 cases. Zhonghua Wai Ke Za Zhi. 2017;55:938–41. 29224270 10.3760/cma.j.issn.0529-5815.2017.12.012

[13] Turk AT Asad H Trapasso J. Mixed corticomedullary carcinoma of the adrenal gland: a case report. Endocr Pract. 2012;18:e37–42. 22548942 10.4158/EP11222.CR

[14] Michalopoulos N Pazaitou-Panayiotou K Boudina M. Mixed corticomedullary adrenal carcinoma. Surg Today. 2013;43:1232–9. 23435808 10.1007/s00595-012-0458-4

[15] Welsh SJ Khan S. Radiological localizing techniques in adrenal tumors. Minerva Endocrinol. 2009;34:161–9. 19471240

